# Changing reference intervals for haemoglobin in Denmark: Clinical and financial aspects

**DOI:** 10.11613/BM.2017.030702

**Published:** 2017-08-28

**Authors:** Judith Ryberg-Nørholt, Henrik Frederiksen, Mads Nybo

**Affiliations:** 1 Department of Haematology, Odense University Hospital, Odense, Denmark; 2 Department of Clinical Biochemistry and Pharmacology, Odense University Hospital, Odense, Denmark

**Keywords:** anaemia, haematology, haemoglobins, reference values

## Abstract

**Introduction:**

Based on international experiences and altering demography the reference intervals (RI) for haemoglobin (Hb) concentrations in blood were changed in Denmark in 2013 from 113 - 161 g/L to 117 - 153 g/L for women and from 129 - 177 g/L to 134 - 170 g/L for men. The aim of this study was to determine the derived change in prevalence of anaemia and the change in yearly health care costs of diagnostic investigations associated with the expected, as we hypothesized, increased prevalence and health care costs.

**Materials and methods:**

Data from 96,314 non-hospitalised patients (55,341 females and 40,973 males, aged 18 - 105 years) from general practitioners and community specialists of Funen, Denmark, were extracted from the laboratory information system. The prevalence of anaemia according to the new and the old RI were investigated, and additional costs were calculated based on estimated additional blood analyses and nationally recommended endoscopic procedures.

**Results:**

Changing the Hb RI increased the number of anaemic patients by 52% (3450 patients) over a two-year period. With new RI the proportion of anaemic elderly above 80 years was 20.5% for females and 43.9% for males. Annual costs of derived additional assessments due to the altered RI were estimated to be 5.7 million €, which equals the cost of 1214 knee replacement surgeries in Denmark.

**Conclusions:**

Changing the Hb RI has been expensive, despite the fact that no outcome studies have justified the alteration. The methodological approach for establishing new RI, here particularly for Hb, should be thoroughly considered. In general, physicians should use RI with caution.

## Introduction

Anaemia is important in clinical medicine. Although a number of different cut-off values for haemoglobin have been suggested, general practitioners often consider a patient anaemic if the concentration of haemoglobin (Hb) in the blood is below the established reference intervals (*1*). Anaemia can be physiological due to pregnancy, it can be due to deficiency of *e.g.* iron, cobalamin or folate, or it can be caused by bleeding, malignancy or chronic illnesses (*2*). Anaemia is associated with poorer outcome of a variety of diseases and previous studies have shown that anaemia increases the risk of hospitalization and mortality in the elderly, mortality in patients with acute ischaemic stroke, and risk of complications due to chronic illness such as kidney failure (*3*-*5*).

The definition of anaemia and thereby the optimal Hb reference intervals has been widely discussed during the last decade, because it is evident that reference intervals must reflect the population for whom they are used. As a result, reference intervals for Hb and how they are established differ between countries. Also, the use of cut-off values has been introduced: a World Health Organisation (WHO) expert committee presented in 1968 a report defining the criteria for diagnosis of anaemia, a report that has been revised in 1989, with cut-offs defining mild, moderate and severe anaemia, and recently addressed in the WHO Vitamin and Mineral Nutrition Information System, where an updated report presents adjustments due to smoking status, residence above sea level, pregnancy and others (*1*, *6*, *7*). Nevertheless, general practitioners generally support their decision-making on the reference intervals given by the laboratory and are not always aware of the different cut-offs or the abovementioned sub-specified classifications. Therefore, the WHO Hb reference intervals have been questioned in multiple cases, initiated by Beutler and Waalen, who in 2006 showed that normal Hb concentrations among healthy adult men and healthy adult non-pregnant women did not fit the reference intervals suggested by the WHO (*6*, *8*). Before the change, these reference intervals had been used for nearly 40 years, and Beutler and Waalen demonstrated that the WHO reference intervals were based on a study with very few data using an inadequate analysis method (*8*).

In 2004, Nordin *et al.* conducted a reference interval study in the Nordic countries: most of the 1826 observations in this study were from Finland and Sweden, while only approximately 13% were from Denmark; the participants were healthy non-pregnant females and healthy males between 18 and 90 years with a mean age of 46 and 48 years, respectively (*9*). The Hb 2.5 - 97.5 percentiles were 117 - 153 g/L for females and 134 - 170 g/L for males. As a consequence Danish Hb reference intervals were changed in 2013 from 113 - 161 g/L to 117 - 153 g/L and 129 - 177 g/L to 134 - 170 g/L for women and men ≥ 18 years, respectively. It is worth mentioning that a similar change has been conducted in many other countries, *e.g.* the United States of America and Germany.

We are not aware of studies that have estimated the impact of altered Hb reference intervals in the general population in terms of increased number of anaemic persons and estimations of the derived health care costs. Furthermore, no outcome studies have been conducted to justify the alteration.

The aim of this study was therefore to elucidate the consequences of changing the Danish Hb reference intervals in terms of anaemia diagnoses and estimated altered economic expenses. Also, we wanted to determine the most likely causes for previously undiagnosed anaemia in order to calculate the fraction of iron deficiency anaemia (IDA) in our population and the hereof derived costs of endoscopic procedures, and to determine if the fraction of IDA was the same before and after the changes of the Hb reference intervals. As the reference intervals were narrowed, we hypothesised an increase in the prevalence of anaemia and an increase in the economic expenses due to more patients in need of additional investigations.

## Materials and methods

In Denmark, general practitioners and community specialists submit their blood samples for analysis at hospital-based laboratories. The Department of Clinical Biochemistry and Pharmacology at Odense University Hospital analyses blood samples for the majority of general practitioners and community specialists on the island of Funen. Funen comprises approximately 381,096 inhabitants ≥ 18 years, corresponding to 8% of the Danish population above 18 years (*10*).

### Subjects

Data from the observed two-year period were extracted from the laboratory information system at Odense University Hospital, containing 214,210 Hb requests either by a general practitioner or a community specialist. All samples were identified by the unique and permanent ID number that all Danes carry. For anonymization these ID numbers were given a temporary unique ID number allowing identification of repeated measurements for the same person. No data were referable to specific individuals and therefore, permissions from Danish authorities were not required.

All Hb measurements were performed on a Sysmex XN9000 (Sysmex Nordic, Copenhagen, Denmark) in whole blood stabilised with tripotassium ethylenediaminetetraacetic acid (EDTA-K_3_). Only the first blood sample with Hb concentration was included, which left 102,137 Hb observations in the data set. Furthermore, 518 observations were missing due to failed analyses and were excluded, and 5305 Hb values for persons under 18 years were also excluded. The final number of Hb observations entering the analysis was 96,314, hereof 55,341 females and 40,972 males aged 18 - 105 years.

For each patient the following analyses results were also retrieved, if they were present within seven days of the included Hb sample: plasma (P)-ferritin, mean erythrocyte corpuscular volume (MCV), P-cobalamin, P-folate, P-creatinine and P-C-reactive protein (P-CRP). Among the included Hb measures, a total of 15,764 P-ferritin, 14,089 MCV, 20,558 P-cobalamin, 9444 P-folate, 86,610 P-creatinine, and 35,029 P-CRP were available.

Observations were categorized by sex and divided into nine age groups. Patients were also divided into groups of “anaemia by old reference intervals” and “anaemia by new reference intervals”. “Anaemia by old reference intervals” was defined as those with anaemia by the old reference interval. “Anaemia by new reference intervals” was defined as those with anaemia by the old reference intervals and the additional number of patients with anaemia by the new reference intervals.

### Anaemia and cost of additional investigations

In order to estimate the additional costs that new reference intervals may generate, we defined a “blood analysis package” for all with anaemia and endoscopic procedures for a selection of patients with iron-deficiency anaemia. The blood analysis package contained measurement of Hb, MCV, mean erythrocyte corpuscular haemoglobin concentration (MCHC), reticulocytes, leukocytes including a differential count, thrombocytes, P-ferritin, P-iron, P-transferrin, P-folate, P-cobalamin, P-lactate dehydrogenase, P-bilirubin, P-haptoglobin, P-creatinine and P-CRP. Costs of these additional analyses alone (9.79 €) as well as the costs of blood sampling (1/30 of a full working day for a laboratory technician, *i.e.* 265.95 €) were estimated to be 18.63 € for the blood analysis package.

Prices for endoscopic procedures were estimated to be 406 € for gastroscopy and 618 € for bidirectional (both gastroscopy and colonoscopy) endoscopy based on Danish national diagnosis-related group procedure hospital rates (*11*). The number of patients in need of bidirectional endoscopy was determined by national guidelines from the Danish Medical Society of Gastroenterology and Hepatology (DSGH): the DSGH guidelines for endoscopic procedures only apply to those with IDA, which involves approximately 50% of all with anaemia in the Danish population (*12*). DSGH guidelines suggest that all patients older than 40 years with IDA should be referred to bidirectional endoscopy due to cancer suspicion. Patients younger than 40 years with IDA should be biochemically screened for celiac disease with measurements of transglutaminase antibodies and IgA. In this age group it was assumed that approximately 4% would turn out positive based on the prevalence of celiac disease in patients with IDA (*13*). Patients younger than 40 years who were biochemically positive for celiac disease were estimated to be referred to gastroscopy alone; however, if biochemically negative they were referred to bidirectional endoscopy under suspicion of cancer, unless they were female and premenopausal which applies to 99% of females younger than 40 years (*14*). Among these patients, IDA was considered physiological without further need of investigation.

To ensure that our data matched the assumption that approximately 50% of all with anaemia in Denmark had IDA, we investigated the frequency of anaemia due to IDA, anaemia of other known causes (AOKC) (categorized as deficiency of cobalamin, folate, or anaemia due to kidney failure), anaemia by chronic disease (ACD) or unclassified anaemia. IDA was defined as follows: patients with anaemia and P-ferritin < 30 μg/L, or patients with anaemia and P-ferritin 30 - 60 μg/L and P-CRP < 10 mg/L, or patients with anaemia and P-ferritin < 100 μg/L and P-CRP > 10 mg/L (*12*). AOKC were defined as patients with anaemia and P-cobalamin < 120 pmol/L and MCV > 103 fL, or anaemia and P-folate < 6 nmol/L, P-cobalamin > 120 pmol/L and MCV > 103 fL, or anaemia and P-creatinine > 150 μmol/L. ACD was defined as P-CRP > 10 mg/L and P-ferritin > 100 μg/L (*12*).

In order to adjust for differences in price levels across countries, additional costs were compared to Danish costs of knee alloplastic surgery: in Denmark, the diagnosis-related group price of one advanced knee reconstruction is 4734 € (*9*).

### Statistical analyses

Normal distribution of Hb values was assessed from histograms for each age group. The 2.5 and 97.5 percentiles were found with 95% confidence intervals (CI) for all patients, patients between 18 and 89 years, patients between 18 and 69 years and for all patients divided into age groups by decade; all subdivided by sex. The proportions of anaemia by the old and new reference intervals respectively were derived for each sex and age group. All data processing was conducted using Stata IC 13 (StataCorp LLC, Texas, USA).

## Results

Table 1 illustrates the distribution of Hb by sex, age, and the 2.5 and 97.5 percentiles for Hb concentrations in these groups. In both sexes the 2.5 percentile was lower for all groups compared to the lower limits for the old as well as the new reference intervals.

**Table 1:** Haemoglobin concentration by age and sex among 96,314 Danish patients from general practitioners

**Age (years)**	**Hb, females (g/L)**			**Hb, males (g/L)**		
	**N**	**2.5 percentile (95% CI)**	**97.5 percentile** **(95% CI)**	**N**	**2.5 percentile** **(95% CI)**	**97.5 percentile** **(95% CI)**
18-105	55,341	103 (103-105)	156 (156-156)	40,973	109 (109-111)	171 (171-172)
18-89	54,124	105 (105-105)	156 (156-156)	40,540	111 (109-111)	172 (171-172)
18-69	41,879	108 (106-108)	156 (156-156)	30,808	122 (121-122)	172 (172-172)
18-19	1137	109 (106-113)	153 (151-155)	580	135 (134-137)	171 (169-172)
20-29	6990	106 (106-108)	153 (153-155)	3514	132 (130-134)	174 (174-175)
30-39	6264	106 (105-108)	153 (153-153)	3684	132 (130-134)	172 (172-174)
40-49	8813	103 (101-105)	156 (155-156)	6128	129 (127-130)	172 (172-174)
50-59	9134	111 (109-113)	156 (156-158)	7850	122 (121-124)	172 (172-172)
60-69	9541	111 (109-113)	158 (158-159)	9052	113 (109-114)	171 (171-171)
70-79	7572	101 (100-104)	158 (156-158)	6782	103 (100-103)	169 (167-169)
80-89	4673	93 (92-95)	156 (155-158)	2950	95 (92-97)	164 (164-166)
≥ 90	1217	89 (87-93)	150 (148-152)	433	85 (74-89)	161 (156-163)
Hb - haemoglobin concentrations. 95% CI - 95% confidence intervals.						

Table 2 shows the proportions of patients with anaemia by the old and the new reference intervals, respectively, categorized by age. Using the old reference intervals 6578 (6.8%) patients were anaemic compared to 10,028 (10.4%) using the new reference interval, *i.e.* an additional 3450 patients with anaemia corresponding to a 52% increase in prevalence of the diagnosis of anaemia. The additional proportion with anaemia ranged between 3 - 9% of all observations in women and 0.5 - 13% of all observations in men across the different age groups. With the new reference intervals an additional 1731 (3.1%) women and an additional 1719 (4.2%) men were defined as anaemic.

**Table 2:** Numbers and proportions of patients with anaemia among Danish patients from general practitioners

	**Women**									
Age (years)	18-19	20-29	30-39	40-49	50-59	60-69	70-79	80-89	≥90	Total
**Anaemia by old reference intervals**										
Anaemia (N)	38	273	285	458	242	258	461	638	283	2936
Proportion within age group (%)	3.3% (2.4-4.6)	3.9% (3.5-4.4)	4.6% (4.1-5.1)	5.2% (4.8-5.7)	2.7% (2.4-3.0)	2.7% (2.4-3.0)	6.1% (5.6-6.6)	13.7% (12.7-14.7)	23.3% (21.0-25.7)	5.3% (5.1-5.5)
**Anaemia by new reference intervals**										
Anaemia (N)	68	469	494	717	397	434	742	959	387	4667
Proportion within age group (%)	6.0% (4.7-7.5)	6.7% (6.1-7.3)	7.9% (7.2-8.5)	8.1% (7.5-8.7)	4.4% (3.9-4.8)	4.5% (4.1-4.9)	9.8% (9.1-10.5)	20.5% (19.4-21.7)	31.8% (29.2-34.5)	8.4% (8.2-8.7)
	**Men**									
**Anaemia by old reference intervals**										
Anaemia (N)	3	41	58	133	309	734	1145	1003	216	3642
Proportion within age group (%)	0.5% (0.2-1.6)	1.2% (0.8-1.5)	1.6% (1.2-2.0)	2.2% (1.8-2.6)	3.9% (3.5-4.4)	8.1% (7.6-8.7)	16.9% (16.0-17.8)	34.0% (32.3-35.7)	49.9% (45.2-54.6)	8.9% (8.6-9.2)
**Anaemia by new reference intervals**										
Anaemia (N)	6	101	107	236	535	1166	1643	1296	271	5361
Proportion within age group (%)	1.0% (0.5-2.2)	2.9% (2.4-3.5)	2.9% (2.4-3.5)	3.9% (3.4-4.4)	6.8% (6.3-7.4)	12.9% (12.2-13.6)	24.2% (23.2-25.3)	43.9% (42.1-45.7)	62.6% (58.0-67.0)	13.1% (12.8-13.4)
Proportions are presented with corresponding 95% confidence intervals.										

Table 3 illustrates that there were 1475 patients with IDA out of 2583 with anaemia and ferritin value for both sexes (57.1% (95%CI 55.2 - 59.0)) with the old reference interval, compared to 1979 patients with IDA out of 3431 with anaemia and ferritin value for both sexes (57.7% (95%CI 56.0 - 59.3)) with the new reference interval. With the new reference intervals 5222 of 10,028 patients with anaemia (52.1% (95%CI 51.1 - 53.1)) were categorized as ”unclassified anaemia”, while only 2983 of 6578 patients with anaemia (45.3% (95%CI 44.1 - 46.6)) were categorized as ”unclassified anaemia” using the old reference interval.

**Table 3:** Anaemia types among the Danish patients sub-group

	**Women**						**Men**					
**IDA**	**AOKC**	**ACD**	**Unclassified anaemia**	**Total anaemia**	**Anaemia + Ferritin value**	**IDA**	**AOKC**	**ACD**	**Unclassified anaemia**	**Total anaemia**	**Anaemia + Ferritin value**
**Type of anaemia for patients with anaemia by old reference intervals**											
Anaemia (N)	1032	444	288	1172	2936	1426	443	849	539	1811	3642	1157
Anaemia by respective type (%)	35.1 (32.2-35.4)	15.1 (13.6-16.0)	9.8 (8.6-10.6)	39.9 (40.2-43.6)	100	-	12.2 (11.1-13.3)	23.3 (22.0-24.7)	14.8 (13.7-16.0)	49.7 (48.1-51.3)	-	-
Anaemia by respective type and ferritin value (%)	72.4 (70.0-74.6)	-	20.2 (18.5-22.5)	-	-	100	38.3 (35.5-41.1)		46,6 (43.7-49.5)	-	-	-
	**Type of anaemia for patients with anaemia by new reference intervals**											
Anaemia (N)	1430	678	393	2166	4667	1970	549	1082	674	3056	5361	1461
Anaemia by respective type (%)	30.6 (29.3-32.0)	14.5 (13.5-15.6)	8.4 (7.7-9.3)	46.4 (45.0-47.8)	100	-	10.2 (9.5-11.1)	20.2 (19.1-21.3)	12.6 (11.7-13.5)	57.0 (55.7-58.3)	-	-
Anaemia by respective type and ferritin value (%)	72.6 (70.6-74.5)		19.9 (18.2-21.8)	-	-	100	37.6 (35.1-40.1)		46.1 (43.6-48.7)	-	-	-
The sub-group comprised 96,314 Danish patients, who had additional blood investigations from general practitioners within seven days of the included Hb sample. Proportions are presented with corresponding 95% confidence interval (CI). IDA - iron-deficiency anaemia. AOKC - anaemia by other known causes (deficiency of cobalamin, folate, *etc.*). ACD - anaemia of chronic disease.												

**Figure 1:**
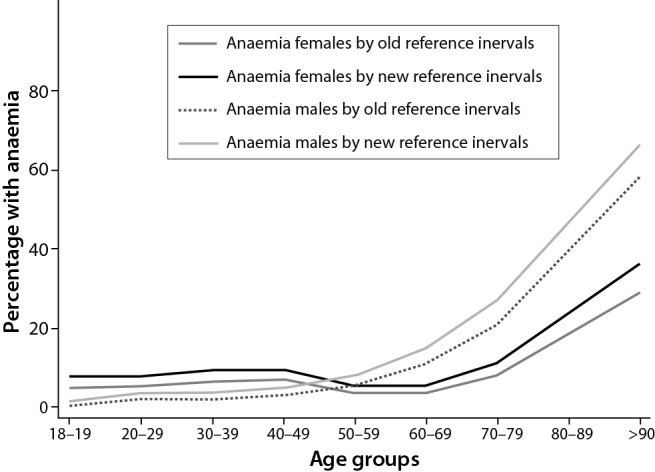
Fraction of anaemic individuals by age for the old and the new reference intervals

As shown in Figure 1, the proportion of individuals with anaemia increased considerably with age for both sexes and with both Hb reference intervals, however with higher prevalence for both sexes using the new reference intervals.

When extrapolated to the entire Danish population, 37,771 patients would be diagnosed with anaemia every year using the old reference intervals, while 57,581 would be anaemic using the new reference intervals. As a result, an estimated additional 19,810 blood analysis packages, 8647 bidirectional endoscopies and 62 gastroscopies would be performed yearly with additional yearly costs of 370,000 €, 5.34 million € and 25,000 €, respectively. Altogether, the additional yearly costs in Denmark due to the change in Hb reference intervals would be an estimated 5.7 million €, equalling the costs of 1214 knee replacement surgeries.

## Discussion

Our study shows that the changed lower reference intervals for Hb possibly increases the prevalence of anaemia by 52%. To our knowledge, this is the first population-based study that reports on this and on the estimated economic consequences of changing Hb reference intervals. As shown, this theoretical increase has the potential to massively increase the costs for anaemia assessment in Denmark with the most pronounced increase in prevalence of the diagnosis of anaemia in the elder age groups. Of note, no public health advantages or disadvantages have been shown to justify the changes or the derived costs, and to our knowledge, no studies have investigated whether changing the Hb reference intervals actually altered long-term morbidity and mortality in this population.

In general, alterations of reference intervals must be scientifically based, in terms of not only underlying demographic data, but also the considerations of what the alterations will lead to in terms of increased/decreased diagnoses, altered treatments *etc*. If the alterations are not evidence-based, they can be expensive and lead to unnecessary and potentially harmful assessments. Studies have shown that the Hb level is affected both by ethnicity and age, and reference intervals should therefore in general not be adopted from other countries or districts without a local validation (*8*, *15*-*17*). The Danish population has a growing immigrant population. In 2014, approximately 11.1% of the adult Danish population were considered immigrants or descendants, and this could affect the prevalence of anaemia due to ethnicity (*10*). As the study by Nordin *et al.* was conducted in 2004 in a Swedish and Finnish population it is likely that the population does not match the Danish population in 2013-2015 (*9*). Furthermore, the study by Nordin *et al.* had a mean age approximately 10 years younger for males and 6 years younger for females compared to our study population. They did not divide individuals into age groups and reported that few cases were older than 75 years. As demonstrated in Table 1 elderly (≥ 70) age groups have a significantly lower Hb 2.5 percentile concentration and thereby a larger proportion of anaemia following this definition. This is also evident from Figure 1 that shows a considerable increase in the prevalence of anaemia already above 50 years of age. However, if a condition is present in almost 50% of the population (for men above 80 years), it could indicate that the definition does not fit the entire population, but rather is defined from studies conducted in a younger population.

Tettamanti *et al.* also described increased anaemia prevalence for elderly over 65 years, where 26.4% of patients with mild anaemia were unexplained while up to one third of the elderly with mild anaemia might be accounted for by myelodysplastic syndrome (*17*). Willems *et al.* found that approximately 30% of elderly with anaemia remained unexplained and found a tendency of lower Hb in elderly with an underlying pathology compared to the anaemic elderly with unexplained anaemia (*18*). As suggested by Merchant and Roy, haematologists needs to address better guidelines for Hb targets for the elderly and must investigate the molecular pathogenesis of anaemia in the elderly more thoroughly (*19*).

The strength of our study is the wide selection of the population over a two-year period. In addition, the use of laboratory data allows the study to be repeated with renewed data. Therefore, it can be used for Hb monitoring in a population at a very low cost. Our study also has limitations. First, the data does not include finger prick Hb measurements from the general practitioners of Funen. Second, the study does not provide any information on causation such as illness, on-going treatment and pregnancies, which could imply selection bias and cause an overestimation of the fraction of patients with anaemia. In order to minimize the number of patients already receiving treatment for anaemia we included only the first Hb measurement. Furthermore, the number of pregnant women with anaemia due to haemodilution could be reduced as pregnant women in Denmark are followed regularly, with the first consult in the middle of the first trimester. At this appointment physicians are encouraged to screen for anaemia (*20*,*21*).

The real expenses in Denmark probably differs from our results as the DSGH guidelines that were revised in 2014 using Hb reference intervals suggested by the WHO (Hb < 120 g/L for females and < 130 g/L for males) (*12*). We did not include diagnostic procedures such as bone marrow biopsies, haemoglobin analyses of stool samples, or fees for extra doctor appointments and special blood samples indicating that our estimated yearly additional costs are likely to be conservative. Finally, we only investigated the consequences of changing the lower limit of the reference intervals. The upper limit was lowered in the current Hb reference intervals; this could increase the number of patients being assessed for polycythaemia. Furthermore, our retrospective data do not take into account that blood sampling has shown a steady increase during the past decades that could increase the costs even more.

Health care budgets are limited and the derived consequences of changing reference intervals must be a part of the decision process in general. To our knowledge, no studies have shown any population benefits of the new, narrower Hb reference intervals. As the prevalence of anaemia is dependent on the prevalence of several diseases it should be considered whether the 2.5 and 97.5 percentile is the best way to determine reference intervals for Hb. Reference intervals based on Hb value limits, where the risk of underlying pathology is significant, might be more cost-effective.

With a generally increasing tendency towards defensive medicine, reference intervals are extremely important parameters when deciding whom to investigate further. It is therefore an important responsibility the decision makers, here the laboratory society, has to carry, namely to assure that alterations such as this are evidence-based and well founded – and to inform the treating physicians if over-diagnosing seems to take place. However, it is necessary for physicians to keep in mind that there can be considerable sex-, age- and ethnicity-related differences in Hb concentrations, and as emphasized in the WHO guideline, to consider other contributory factors, *e.g.* smoking and sea level (*7*). Therefore, the diagnosis of anaemia should always be decided in context with other blood analyses and clinical symptoms. Of importance, extensive diagnostic programs should never be based on a single finding of mild anaemia without symptoms.

In conclusion, changing the Hb reference intervals increased the prevalence of the diagnosis of anaemia by 52%, which may increase heath care expenditures considerably due to additional investigations. Whether this actually happens, and what impact it will have on morbidity and mortality warrants further investigations.
